# Haplotype-defined linkage region for gPRA in Schapendoes dogs

**Published:** 2007-02-07

**Authors:** Tanja Lippmann, Anna Jonkisz, Tadeusz Dobosz, Elisabeth Petrasch-Parwez, Jörg T. Epplen, Gabriele Dekomien

**Affiliations:** 1 Human Genetics, Ruhr-University, Universitätsstraße 150, 44801 Bochum, Germany; 2 Medical University, Ul. Curie-Sklodowskiej 52, 50-369 Wroclaw, Poland; 3 Neuroanatomy and Molecular Brain Research, Ruhr-University, Universitätsstraße150, 44801 Bochum, Germany

## Abstract

**Purpose:**

In order to determine the molecular basis of canine generalized progressive retinal atrophy (gPRA), we initiated whole-genome scanning for linkage in gPRA-informative pedigrees of the Schapendoes breed.

**Methods:**

Detailed pedigree and ophthalmological data were assembled in selected Schapendoes pedigrees. A whole-genome scan was initiated by two-point linkage analysis using microsatellite markers in combination with haplotype analyses. Mutation screening was carried out in respective candidate genes by DNA sequencing of amplified products and quantitative real-time reverse transcriptase polymerase chain reaction (RT-PCR).

**Results:**

Genotyping data of the microsatellite genome scan evidenced a peak two-point lod score of 4.78 for marker REN93E07 on CFA20. Haplotype analyses inferred the gPRA locus in a 5.6 megabase (Mb) region between markers FH3358 and TL336MS. Mutation screening in the genes *CACNA2D3*, *HT017*, and *WNT5A* revealed no causative sequence deviations. In addition, *CACNA2D3* mRNA levels were equivalent in retinas of affected and healthy dogs.

**Conclusions:**

By genome-wide linkage analysis a region for gPRA was identified and fine-localized in Schapendoes dogs. Although the mutation causing gPRA in Schapendoes dogs has not yet been identified, we established indirect DNA testing for gPRA in this breed based on linkage analysis data.

## Introduction

Generalized progressive retinal atrophies (gPRAs) in domestic dogs (*Canis familiaris*) are a group of inherited retinal dystrophies that share a similar phenotype. gPRA causes progressive loss of vision, usually leading to blindness. Initially rod photoreceptor vision is affected, causing night blindness followed by progressive loss of cone photoreceptors with deteriorations in daytime vision. gPRAs can be classified by age of onset and rate of progression [1]. Certain breeds show early onset forms, e.g. Collies, Irish Setters, Norwegian Elkhounds and Miniature Schnauzers. In these breeds, the disease results from abnormal or arrested development of the photoreceptor cells in the retina, and gPRA affects pups very early in life. In other breeds (including Miniature Poodles, English and American Cocker Spaniels, Labrador Retrievers) gPRA onset occurs much later. Affected dogs in these latter breeds appear normal when young, but develop gPRA as adults.

Two X-linked [2] and an autosomal dominantly inherited trait [3,4] have been described, yet most gPRA forms are transmitted in an autosomal recessive (AR) mode. Up to now, causative mutations have been identified only in few breeds of dogs with AR transmitted gPRA [5-11].

In addition to the respective pedigree material highly informative polymorphic DNA markers [12,13] are the necessary tools for mapping chromosomal locations of disease gene loci by linkage analysis. In order to determine the molecular basis of canine gPRA, we initiated whole-genome scanning (WGS) using markers spread evenly across the canine genome for linkage in gPRA-informative pedigrees of the Schapendoes breed. Here we demonstrate linkage of the gPRA trait to markers on canine chromosome 20 (CFA20) in Schapendoes. In addition, the critical region was fine mapped, and the novel candidate genes *CACNA2D3*, *HT017*, and *WNT5A* were investigated.

## Methods

### Animals

All dogs were collected from the general breeding population of pure-bred Schapendoes. Five pedigrees, comprising 57 Schapendoes dogs including 13 gPRA-affected animals, were available in which gPRA is transmitted in an AR manner. Ophthalmologically experienced veterinarians confirmed the gPRA status of affected and unaffected dogs by ophthalmoscopy as documented in certificates of eye examinations. gPRA in Schapendoes is characterized by late onset and slow progression as documented by veterinarians of the Dortmunder Ophthalmologenkreis (DOK). Affected Schapendoes dogs appear normal when young, but develop gPRA at an age of onset between 2-5 years. Early in the disease, affected dogs are night-blind, lacking the ability to adjust their vision to dim light; later, their daytime vision also fails. This process of complete photoreceptor degeneration takes up to 2 years.

Genomic DNA was extracted from peripheral blood according to standard protocols [14]. For isolation of RNA and retina sections we obtained an eye of a gPRA-affected, five year-old Schapendoes with complete loss of night vision yet anecdotally remaining, very limited day-time vision. Retinas of gPRA-free Saarloos/Wolfshounds were used as controls.

### Histology

The enucleated eyes from a five year old, gPRA-affected Schapendoes dog and control eyes from a gPRA-free Sarloos/Wolfshound were sagittally cut at the level of the optic nerve, immersion-fixed in 100% ethanol and paraffin-embedded. Serial sections, 15 μm thick, were cut over the whole extension of the retina, stained with hematoxylin and eosin and photo-documented.

### Markers and genotyping

**Figure 1:**
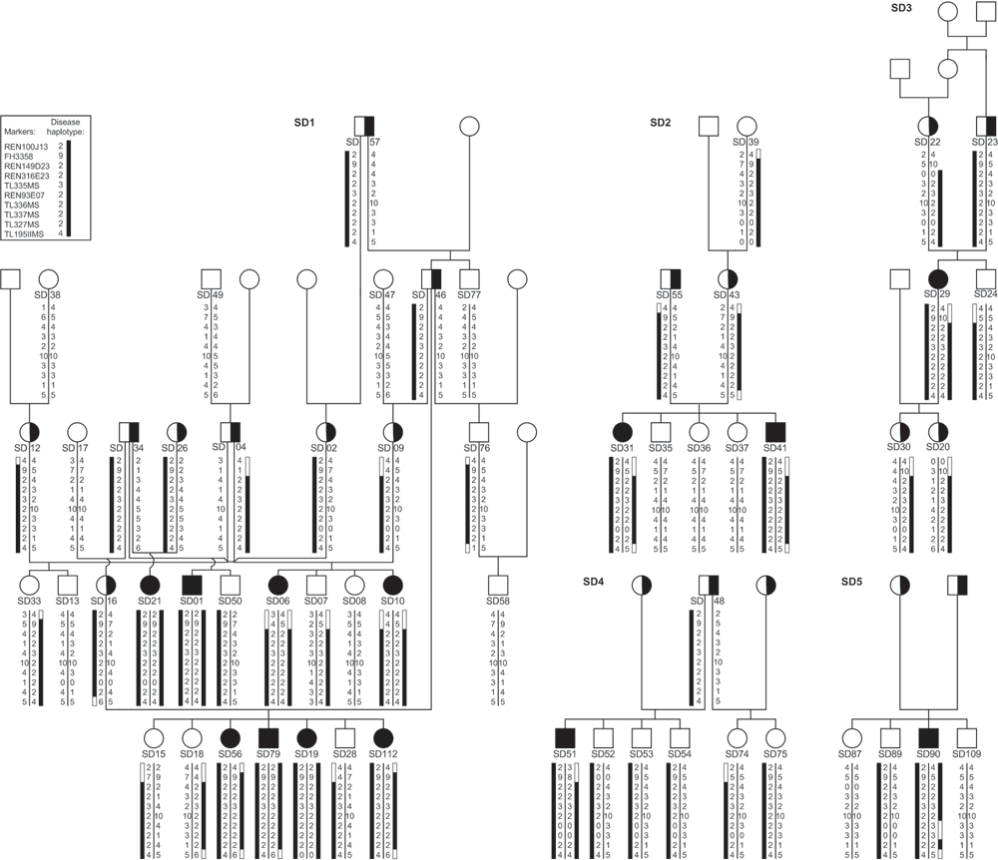
Haplotypes of the gPRA Schapendoes families SD1-5 as established by microsatellite markers for chromosome 20. Affected dogs are represented by black, unaffected by white and those with known carrier status are represented by half-filled symbols. Circles represent females and squares represent males. Genotype that could not be ascertained are scored as "0". Black bars indicate the affected haplotype. In the box, genotyped markers and the disease haplotype are indicated. The observed recombination events evidence the disease causing locus in the region between markers FH3358 and TL195IIMS.

For the WGS highly informative autosomal microsatellite markers were analyzed from the minimal screening set 2 (MSS-2) [12]. Microsatellites for fine mapping (Table 1, Figure 1) were identified using published dog markers [15], the dog genome sequence (May 2005) and the Tandem Repeats Finder included in the UCSC Genome Browser. Only microsatellites with a repeat length exceeding 15 units were selected. PCR primers were designed using Primer Express software (PE Biosystems). For PCR we used the "tailed primer PCR" as described before [16]. This method requires three oligonucleotides for amplification: 1. tailed forward primer (tailed F), 2. reverse primer and 3. labeled primer (labeled F) corresponding to the 5'-tail sequence of tailed F. PCR conditions were as follows: 1-PCR buffer (Genecraft, Lüdinghausen, Germany), 0.2 mM each dNTP, 1.5 to 3 mM MgCl_2_, 0.2 pmol tailed F, 2.5 pmol labeled F, 2.5 pmol reverse primer, 0.5 U BioTherm DNA Polymerase (Genecraft) and 50 ng DNA. PCRs were performed in 96-well microtiter plates (Thermowell Costar Corning, NY). Each well contained a reaction volume of 10 μl. A "touchdown" PCR procedure was applied in a thermocycler (Biometra, Göttingen, Germany): initial denaturation (5 min at 95 °C), two initial cycles 6 °C and 3 °C above the annealing temperature, 40 cycles of 95 °C (30 s), annealing temperature of 53 °C (30 s), elongation at 72 °C (30 s) and a final elongation step at 72 °C (3 min).

**Table 1:** Microsatellite markers for mapping, primers for PCR amplification, their location and PCR products sizes.

**Marker**	**Microsat type**	**Primer sequence (5'-3')**	**Location on CFA20**	**Size (bp)**
REN100J131	di	TGATTGACTCTACTTTACACA	25,817,177	164
		TATATTAGGCGGTTTTCTTCT	25,817,034	
FH33582	tetra	CATCACCCAAATTCAAAGGCA	33,096,985	268
		CCATCAAGGCCCTAATATTTAAAGATT	33,097,252	
REN149D232	di	GACAGAAGAGCCCATCGAG	37,609,209	168
		CATAGTCACACCCACCAATG	37,609,376	
REN316E232	di	AAAAAGAGGATGGGATGGAG	38,133,160	154
		TCAGATAGATCATTTGCTGCC	38,133,313	
TL335MS	di	CCCCATAGAAAAGGGACTG	38,313,482	126
		CACTTTCTCTCCCCCTCTG	38,313,607	
REN93E071	di	GGCCCCCTCACCACTCC	38,525,708	170
		TGAGGGCTGCCACTGTAAATA	38,525,877	
TL336MS	di	TCACTGGTACAGGCATTGTTC	38,727,171	133
		CCTTATGTCCATCCCCATC	38,727,303	
TL337MS	di	AAGGCTACTTTTGGGACCC	38,956,429	335
		TGAGAGGTGAGAGATGCTGG	38,956,763	
TL327MS	di	TGGCTTGTTATGAAGTTGGCC	39,812,660	167
		AGCCCCAGGTGCTATGGAG	39,812,826	
TL195II	di	AACTGAGGTTCCCTTGTTCC	47,049,704	232
		CTAATCGAAAGTGCAGGAGG	47,049,935	
REN114M191	di	CCATACAGCCACACCAAGTG	56,553,756	192
		GCTCCCTGACCACAGGTCT	56,553,948	

Location ascertained from UCSC Genome Browser (assembly: dog May 2005).

Electrophoreses were run using Amersham Biosciences standard protocols for genotyping on the automated capillary DNA sequencer (MegaBACE 1000, Amersham Biosciences, Freiburg, Germany). PCR products of two markers were diluted (each 1:10), pooled and 2 μl of this dilution were mixed with 0.5 μl of MegaBACE^TM^ ET-R Size Standard and 2.5 μl loading solution (70% formamide, 1 mM EDTA). Raw data were analyzed by the MegaBACE Fragment Profiler software version 1.2 (Amersham Biosciences, Freiburg, Germany) and a table of genotypes was exported. For a few markers no PCR products were obtained for specific dogs.

### Linkage analyses

Using the LINKAGE package version 5.1 [17], we undertook two-point linkage analyses between the gPRA locus and each marker. The disease trait was coded as being AR transmitted with full penetrance, no phenocopy and a frequency of 0.1 for the disease allele. The data were controlled for Mendelian inheritance using the unknown program, and two-point linkage analyses were performed using mlink program [17]. Marker-allele frequencies were calculated on the basis of genotype data of 10 unrelated individuals of the schapendoes breed.

### Mutation screening of candidate genes *CACNA2D3*, *HT017*, and *WNT5A*

Exon/ intron boundaries of the canine genes *CACNA2D3*, *HT017*, and *WNT5A* were searched for by comparison of the mRNA sequence of the human gene (accession numbers: *CACNA2D3*, NM_018398;*HT017*, NP_065729;*WNT5A*, NM_003392) with the publicly available dog genome sequences in UCSC Genome Browser (assembly: dog May 2005). For sequencing of the coding regions of the three genes, intronic PCR primers flanking the exons were designed in order to amplify at least 50 intronic bases on either end of the exon in order to cover the splice junctions (Table 2). PCRs were performed under standard PCR conditions [18] with BioTherm DNA Polymerase (Genecraft) and 1.5 mM MgCl_2_ at an annealing temperature of 57 °C.

**Table 2:** Primers for amplification of exonic regions of *CACNA2D3*, *HT017*, and *WNT5A* including exon/intron boundaries.

**Gene**	**Exon**	**Sequence (5’-3’)**	**Size (bp)**
CACNA2D3	1F	GGTGCTGGCAGTTTCTACCC	268
	1R	ATCGCTTCCTGCCACCAC	
	2F	AAGAGAGGCAGTCCTATTTATTCCTTG	329
	2R	GAACAACTGAGTCAACTTCACTCTTTG	
	3F	AAGGCTCCAGGTCATAATGCAC	261
	3R	CTTACCATTTCTTGTTTGGCAGC	
	4F	GACACCCTTACCTGGCTTTGC	355
	4R	TGTCCCCCATACCCTCTGTTC	
	5F	CTCATGCTGCTATTGCCAGC	326
	5R	TCATTTTCCCCTATTGGGAGG	
	6+7F	CAGGGCTGGTCATTGCATG	546
	6+7R	AGGCGGGACATCTAATGCTG	
	8F	GGTACCCCTCAAATGATTAATTGTG	201
	8R	TAGGTTTGTATCATGACCCTTGATG	
	9F	CCATTCCAGTCTGAGACTCGTTG	311
	9R	GCCAAGGCTAACCATAATGCTG	
	10F	GGGAAATGCTTATCGTGTAGGTTC	422
	10R	AGAGCCATAGTATTCGGTTTGGTC	
	11F	CCTACAGATGGGAAGCCTGG	277
	11R	CTCTTAACTATCCCAACTTGCGC	
	12F	TGAGAGGTGGGTTTATGGTTGAC	394
	12R	GGTTAACACGGTCTTCTGGAATTAAC	
	13F	TTGCCAGTGGTGACCAAATG	208
	13R	GTACTGAATGCACAAAATCATGGAG	
	14F	ACTGGTCTGTTCCCAATGGC	266
	14R	CACCAGAGCTTGAGTCAGAGAATG	
	15F	AAACTTTGAGAAGGCACCAGATG	248
	15R	AGGTATCTTGGACGCTTAGTCCTG	
	16F	TGAATTCACCAGGGAGCCAG	221
	16R	AAACGCTGCTGCAAAAGTGTC	
	17F	GCTTCTCTCCAATATCCCTCCAC	279
	17R	TGGCCTCCAGGTACAGAACTG	
	18+19F	AGCATGCTGACTTGGTGCC	693
	18+19R	CACTCTTTCCAGCCTTTTGGC	
	20F	AAACCAACACCAACTATAATCAGTGG	310
	20R	TCTTTCATTAATCCAATGACTGGC	
	21+22F	GGGAGATGCAAGTGAGGAGG	637
	21+22R	GCTGGATTTGATGGGACCG	
	23F	AGAACTGTTCCATATTTTCAGCTGG	406
	23R	TCGAGCTCTTGTGGTTTGGAG	
	24F	TTGCATTCTGTGCTTGGTGC	359
	24R	ATCAAGAAACGCAAGCCTCC	
	25F	TCTCCACACTTTCGCCAACAC	484
	25R	GGCCAATATTCAACACTGCCTC	
	26F	CACAGCAGCTATGGCGTCAC	410
	26R	ACCAGCTGTCAGAATCTTGAATTG	
	27F	TGCTTGCATCAGCTTCTTGC	251
	27R	GCACTTTTAGGCTCCGGGTAC	
	28F	TAAGTGGCAATCATAGCAGATGC	232
	28R	TCAGGAGCAATCTGTCAGACAAG	
	29F	CCATGGCCTTAACTCCTAGAGC	381
	29R	GGCTGTGACATTTTTAGAGGGATAG	
	30F	TCCTGATTCTTCTCTGGTGCTTG	231
	30R	GGCTTGCTTTATTTGACCTTGG	
	31F	GACTTTGCATGTTCCTGGTTTG	304
	31R	CACAGATAAAGATATGCTCACGCTC	
	32F	GGTATCCAACCATGTTCTTCTTGTAG	373
	32R	TGGTCTCAGCTTTCAATATTTCGAG	
	33F	GGGTAACACAACCAACCATACTGTAC	290
	33R	TGTATGTTTGTCCATCCTTGGTG	
	34F	GAGCGGAACTGGGTTCTGAG	352
	34R	TCTAGCGAAGCCAAATGATGC	
	35+36F	GGGAGTTTTCCTCAGGATCTTTG	551
	35+36R	ATGAGACTCTCATGGTGAATCTGG	
	37F	AAGACTTGATTCCTACTTTGGAGAGC	630
	37R	AAGTCTCTGCCAACAACCATCC	
HT017	1F	GCGTTGAAGAAAAGCACAAGC	209
	1R	AGTCCCTTCCTCCCACGG	
	2aF	ATTCCTTGTGACTTCAGAGGGAC	487
	2aR	GGAGCTTGATTTGTCATCAAACA	
	2bF	GCAGACTTTTCCACTCCCTCC	319
	2bR	CCTGCGTGTTACCAGCCTAGT	
	3F	GCTGTATATATTCCATCTGCCTGAG	605
	3R	GTGAATGCATCAACCCTACTCATATAC	
WNT5A	1F	GTAAAGTCTTTTGCACAATCACGC	468
	1R	AAAAAGTGGCGAGCGTCG	
	2F	AACTCAACGGAGGAGAAGCG	302
	2R	AATAAAACAAAGCATATGTACTTAGAAGGAAC	
	3F	ACTTTGTCATGAGGACAAGCAGG	440
	3R	TCTTCAGGAGAACACTTGATCCG	
	4F	GGGTCAGAGTGGAGACGCC	503
	4R	TTGTCAGGCAGCATCAGGC	
	5F	GGAGCGGAGCTTTGGTAACC	599
	5R	AAATAAGTGGGTCCTGGGAGC	

The intronic primer sequence for amplification of corresponding exons and 50 intronic bases on either end of the exons and the product sizes are shown.

For sequencing of the *CACNA2D3* cDNA total RNA of retinal tissue was isolated. For this purpose peqGOLD TriFast reagent (Peqlab, Erlangen, Germany) was added to the frozen tissue samples, the mixture was immediately homogenized and total RNA was then extracted using guanidinium isothiocyanate (RNeasy Mini Kit, Qiagen, Hilden, Germany). cDNA was synthesized by oligo-dT priming with the Sensiscript RT Kit (Qiagen, Hilden, Germany). Overlapping PCR products of the cDNA of the *CACNA2D3* gene were generated using the primers in Table 3.

**Table 3:** Primers for amplification of *CACNA2D3* cDNA.

**Primer**	**Sequence (5'-3')**	**Size(bp)**
CACNA2D3 Ex2 F	ATGGAAGAGATGTTTCACAAAAAGTC	605
CACNA2D3 Ex7 R	GCAATAATGTTGAAAAAGTCATCATCC	
CACNA2D3 Ex6 F	ATTAAATGGGAACCAGATGAGAATG	375
CACNA2D3 Ex9 R	ATCACTGAGAATGTTGAAGGCC	
CACNA2D3 Ex9 F	CACTTCAGGGAGCATCTGGAC	728
CACNA2D3 Ex17 R	CACTCCACCTCAGAGAGGTCAAC	
CACNA2D3 Ex15 F	AGATCGAAAGGCATTCTTCTGG	624
CACNA2D3 Ex23 R	GGCATCAAAGAGGACTTCTTGTATC	
CACNA2D3 Ex20 F	AGGTGTGGCGCTCTCCAG	752
CACNA2D3 Ex29 R	CCAGAATAAACCCATTATTGTCTATGAG	
CACNA2D3 Ex28 F	GCCTCTCTGGATGGCAAATG	762
CACNA2D3 Ex37 R	TCACCTTGAGAAGAGCATTAAGAGC	

The exonic primer sequences for amplification of overlapping polymerase chain reaction products of the cDNA and the product sizes are shown.

All sequencing reactions were carried out by the dideoxy chain termination method using the Dyenamic ET Terminator Kit (Amersham Biosciences, Freiburg, Germany) according to the manufacturer's instructions. Reaction products were run on an automated capillary DNA sequencer (MegaBACE 1000, Amersham Biosciences, Freiburg, Germany).

### Quantitative real-time RT-PCR of candidate gene *CACNA2D3*

Total RNAs from the retinae of a gPRA-affected Schapendoes and an unaffected Saarloos/Wolfshound were subjected to quantitative real-time RT-PCR analysis using the QuantiTect SYBR Green assay (Qiagen, Hilden, Germany) as described by the manufacturer and the iCycler iQ real-time PCR detection system (Bio-Rad, München, Germany). PCR primers were designed using Primer Express software (PE Biosystems). In order to avoid amplification of contaminating genomic DNA, the primers span an intron. *CACNA2D3* mRNA/cDNA was amplified using primers CACNA2D3 Ex9-F (5'-CAC TTC AGG GAG CAT CTG GAC-3') and CACNA2D3 Ex10-R (5'-GGC TGC AGA TGC TTC CTTG-3'). ATP-binding cassette, sub-family A, member 4 gene (*ABCA4*) and guanine nucleotide binding protein, α transducing activity gene (*GNAT1*) are retina specific. They were amplified using primers ABCA4-F (5'-TGG AGG AAA GCT CCC AAT CC-3') and ABCA4-R (5'-GCC TCT CTG GTG ATA GGG CC-3') and GNAT1-F (5'-GCT CGC GTG TCA AGA CCA C-3') and GNAT1-R (5'-ATC CAC TTC TTG CGC TCT GAG-3'). Hypoxanthin phosphoribosyltransferase 1 (HPRT1) served as internal reference and was amplified using primers HPRT1-F (5'-AGC TTG CTG GTG AAA AGG AC-3') and HPRT1-R (5'-TTA TAG TCA AGG GCA TAT CC-3'). One-step PCR cycling was carried out by reverse transcription at 50 °C for 30 min, initial activation step at 95 °C for 15 min x1 cycle, 4-step cycling at 94 °C for 15 s, at 60 °C for 30 s, at 72 °C for 30 s x 40 cycles. As soon as the PCR was completed, baseline and threshold values were set automatically and threshold cycle (CT) values were calculated. CT values were exported to Microsoft Excel for calculating the real copy number. The CT value represents the PCR cycle at which an increase in fluorescence can first be detected above a base line signal level. Signals were quantified by normalizing with GAPDH expression.

## Results

**Figure 2:**
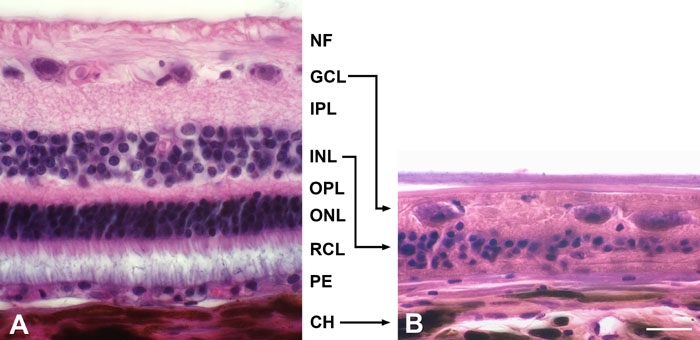
Hematoxylin-, eosin-stained paraffin sections illustrating normal canine retina and gPRA-affected retina of a Schapendoes dog. **A**: The retina of a Saarloos/Wolfshound exhibited regular nuclear layers with the nerve fiber layer (NF), ganglion cell layer (GCL), inner plexiform layer (IPL), inner nuclear layer (INL), outer plexiform layer (OPL), outer nuclear layer (ONL), photoreceptor layer (RCL), and the pigment epithelium (PE). **B**: In the degenerated retina of a Schapendoes dog the outer retina with the RCL and ONL was missing, the INL and the IPL reduced, the GCL was comparatively preserved. CH represents choroid. The scale bar in **B** represents 20 μm; same magnification for **A** and **B**.

Compared to a normal retina (Figure 2A), the gPRA-affected eyes of a five year old Schapendoes displayed typical degeneration signs in peripheral and central areas (Figure 2B). The outer retina with the photoreceptor layer and the outer nuclear layer was missing in all retinal parts investigated. The inner retina showed reduced inner nuclear and inner plexiform layers, whereas the ganglion cell layer appeared comparatively preserved.

A detailed ophthalmological examination was performed on all 57 Schapendoes of five different families revealing 13 dogs with bilateral affection. Evaluation of gPRA in these five families suggested that the disease segregates as an AR trait (Figure 1). A case of inbreeding was identified in family SD3. Initially, WGS for linkage was performed in the five pedigrees using markers of the MSS-2 [12]. Having completed the typing of 165 microsatellites (CFA1-20 and 24-26) of 325 MSS-2 markers, the WGS was abbreviated after a two-point lod score of 4.78 at q=0.000 was obtained for marker REN93E07. The high lod score indicated that the gPRA phenotype was linked to a mutation on CFA20. For fine mapping of the gPRA region in the Schapendoes breed nine additional microsatellite markers from CFA20 were genotyped between marker REN100J13 and TL195IIMS (Table 1, Figure 1) in the five pedigrees. With one exception all microsatellites represent dinucleotide repeats. Heterozygosity values range around 0.5, and 4-10 different alleles were analyzed in the Schapendoes population. Two-point lod scores for linkage between the gPRA locus and microsatellite markers gave still a maximum lod score of 4.78 with marker REN93E07.

**Figure 3:**
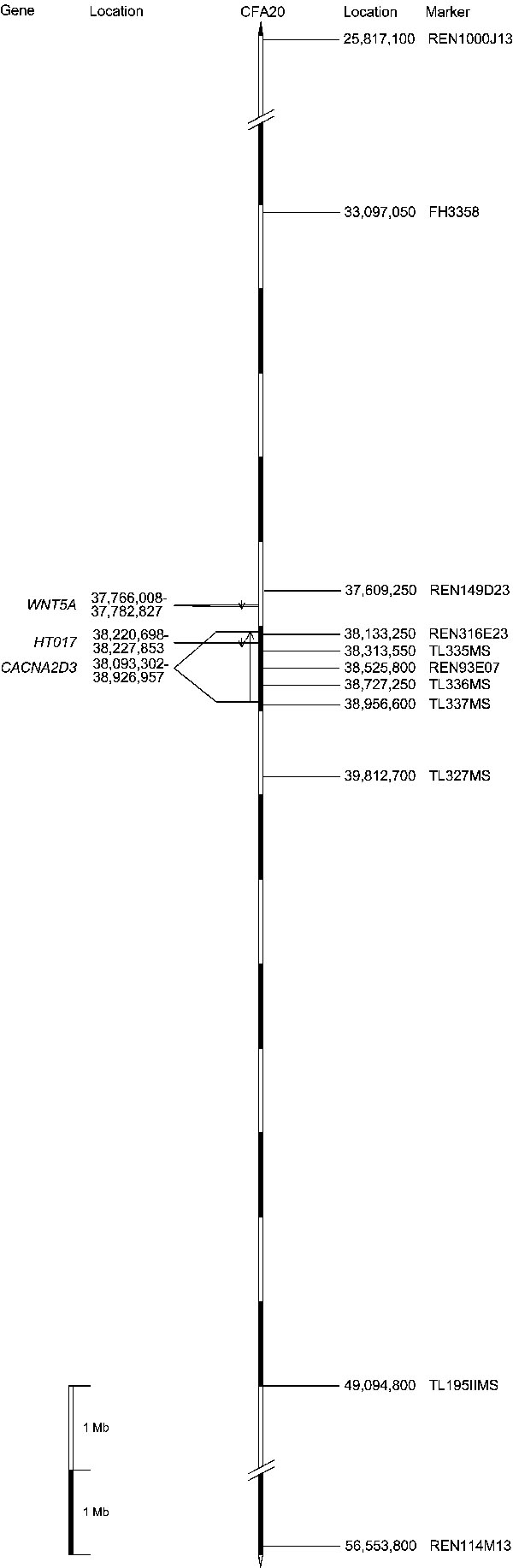
Schematic overview of the critical region on chromosome 20 in which the gPRA locus maps in Schapedoes dogs. On the left hand side of the chromosome the analyzed genes and their genomic location are shown, on the right hand side genotyped microsatellite markers are depicted. Location ascertained from UCSC Genome Browser (assembly: dog May 2005).

Genotyping of ten microsatellite markers for all 57 dogs of the five pedigrees revealed that the "2-2-3-2-2-2-2" haplotype (marker REN149D23 to TL327MS) segregates with the gPRA trait and has a frequency of 50% in the analyzed pedigrees (Figure 1). Analysis of this haplotype placed the gPRA locus in a region between marker FH3358 and TL195IIMS. A potential double recombination event in the dog SD90 of family SD5 may be interpreted to confine the size of the critical haplotype to 5.6 Mb flanked by markers FH3358 and TL336MS. For the "recombined region" of dog SD90 no canine gene has been published in the different gene banks so far. Comparison of the canine DNA sequence of the critical region with the human genome in UCSC Genome Browser (assembly: human May 2004) shows homology with chromosome 3p. In man this region comprises candidate genes for retinitis pigmentosa (RP)-and thus also for gPRA in Schapendoes. The marker with the peak lod score REN93E07 is located in intron 7 of candidate gene *CACNA2D3* (Figure 3) which spans a genomic region of about 830 kb (kb) and consists of 37 exons. The mRNA of this gene encodes the calcium channel α_2_δ_3_ subunit, which is mainly expressed in brain [19] and also in the eye UniGene. In intron 26 of the human *CACNA2D3* gene the *HT017/LRTM1* gene is located (Figure 3). This gene encodes the leucine-rich repeat and transmembrane domain 1. Upstream of *CACNA2D3* the *WNT5A* gene is situated (Figure 3) encoding member 5A of the wingless-type MMTV integration site family. Sequencing of the candidate genes *HT017* and *WNT5A* did not reveal any pathogenic mutations, neither in the coding sequences nor in intron/exon junctions of affected individuals. Five single nucleotide polymorphisms (SNPs) were detected in the *CACNA2D3* gene: IVS16-7T>C, IVS23-51A>T, IVS29+18A>G, IVS29+57C>T, IVS30-57T>C. Yet, these SNPs occur in homozygous state not only in gPRA-affected dogs, but also in healthy Schapendoes (data not shown). Thus these SNPs do not cause gPRA in Schapendoes. Additionally, a thymin-insertion in intron 6 (IVS6-38_-34insT) was identified in comparison to the UCSC dog genome sequence (assembly: dog May 2005). Further investigations revealed that this insertion was present in homozygous state in healthy dogs of other breeds, implying a non-pathogenic polymorphism. Furthermore, sequencing of the *CACNA2D3* cDNA from a diseased Schapendoes eye revealed no sequence deviations, thus excluding "hidden" mutations affecting the splicing process. In addition, altogether 25 kb in introns 5, 7 and 8 of the *CACNA2D3* gene comprising evolutionarily conserved sequences were analyzed without any hint on the gPRA mutation in question (data not shown).

**Figure 4:**
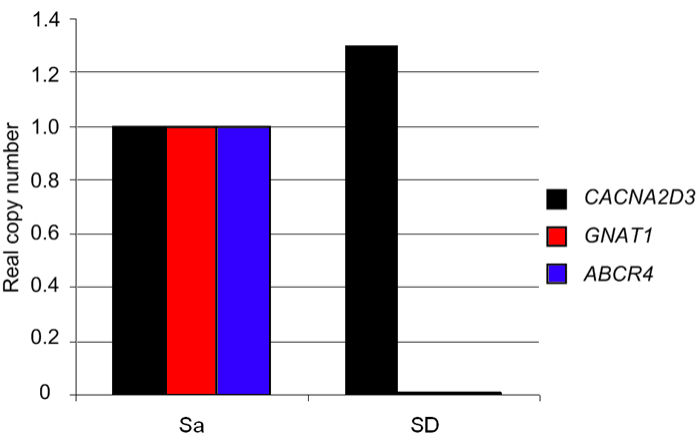
Analysis of gene expression by quantitative real-time reverse transcriptase polymerase chain reaction. Real-time RT-PCR was used to determine the expression of the genes *CACNA2D3*, *GNAT1*, and *ABCA4* by calculating the real copy number. Expression levels were normalized to those of the *HPRT1* gene. No differences were obvious between the unaffected Saarloos/Wolfshound (Sa; N=2) and the g-PRA affected Schapendoes (SD; N=1) for *CACNA2D3* mRNA. *GNAT1* and *ABCA4* mRNA expression is substantially reduced or absent in retinal tissue of the affected Schapendoes.

In order to exclude potential transcriptional impact of the elusive gPRA mutation, the expression of *CACNA2D3* mRNA in retinal tissue was determined by real-time RT-PCR (normalized to the level of the housekeeping gene *HPRT*). mRNA expression of two unaffected Saarloos/Wolfshounds was compared to a gPRA-affected Schapendoes. *GNAT1* and *ABCA4* gene expression was substantially reduced in retinal tissue of the affected Schapendoes. In contrast, no reduction was obvious for the *CACNA2D3* mRNA levels (Figure 4).

## Discussion

The responsible locus for gPRA in Schapendoes, a canine counterpart for RP in man, maps to the central region of CFA20. Haplotype analyses defined the critical interval between marker FH3358 and TL195IIMS. The haplotype potentially confining a smaller critical interval (between FH3358 and TL336MS) occurred exclusively in individual SD90 of family 5. A caveat remains to accept this confinement: Alleles 3 for markers TL336MS and TL337MS might not represent a recent or ancestral recombination event, but rather be due to non-mendelian inheritance. Yet non-mendelian inheritance appears less likely for two adjacent microsatellites in the absence of any additional slippage mutations of these microsatellites in all the other analyzed meiotic events. Consequently, we concentrated initially on the smaller critical interval between FH3358 and TL336MS, albeit the genomic region between TL337MS and TL195IIMS must not be excluded formally to comprise additional candidate genes.

An interesting candidate in the critical genomic interval represents the *CACNA2D3* gene. The marker with the highest lod score REN93E07 was located in intron 7. Direct sequencing of all coding exons for homozygously affected and normal SD dogs exclude a gPRA-causing mutation in the coding sequence of the *CACNA2D3* gene. This fact implies that the mutation causing gPRA in Schapendoes may be located intronically in the *CACNA2D3* gene affecting splicing. Yet, extensive sequence analysis of retinal cDNA revealed no splice mutation in the *CACNA2D3* gene. Furthermore, in order to exclude the *CACNA2D3* gene as causative for gPRA in Schapendoes, we analyzed the expression of *CACNA2D3* mRNA in retinal tissue: mRNA levels were nearly identical between a gPRA-affected Schapendoes and an unaffected Saarloos/Wolfshound. In contrast, the expression of the retina-specific genes *GNAT1* and *ABCA4* appeared reduced substantially or even absent. Photoreceptor cells in the retina of the affected Schapendoes have vanished so that an expression of retina-specific genes cannot be demonstrated. Since its mRNA expression is unaltered in a retina without photoreceptor cells, the *CACNA2D3* gene appears expressed mainly in the cell types not affected by gPRA. Yet also photoreceptor cells may well produce small but crucial amounts of *CACNA2D3* transcripts that do not significantly affect the mRNA levels from unseparated retinal extractions. Given the availability of respective tissue samples, we could nevertheless use the haplotype-defined linkage region data to examine obligatorily homozygotic mutation carriers for altered retinal mRNA expression already presymptomatically.

Since gPRA in Schapendoes is probably not caused by a *CACNA2D3* mutation, mutation analysis of the *HT017* and *WNT5A* genes were performed. Yet, gPRA-causing mutations were excluded in the coding sequence of these two genes. In comparison to the UCSC dog genome sequence (assembly: dog May 2005) the critical region between marker FH3358 and TL336MS comprises further candidate genes, which are be investigated. In case the causative mutation is not identified in this critical interval, additional candidate genes are to be investigated in the region between TL337MS and TL195IIMS. Although the mutation causing gPRA in Schapendoes has not yet been identified, the critical region for the location of the mutation was reduced to 5.6 Mb. Our findings for gPRA in the Schapendoes breed constitute an interesting naturally occurring model for RP, the human counterpart of gPRA.

Based on linkage analysis data, we established an indirect DNA test for gPRA in this breed. Nearly 600 dogs with a mean age of five years have been tested so far. Based on the age of onset of 2-5 years in the Schapendoes breed, affected dogs among the tested individuals are likely to show initial gPRA symptoms. All 18 phenotypically affected dogs were tested as harbouring the linked haplotype in homozygous state, and all healthy obligatory carriers were typed as being heterozygous. The degree of certainty for a test result depends on the rate of recombination (so far no recombinations were observed) or new mutations. Theoretically small uncalculatable risks remain for false negative and positive results, respectively. Notwithstanding, the established indirect DNA test facilitates the eradication of gPRA among Schapendoes. Known mutation carriers can still produce offspring by selective crossing to dogs with mutation-free haplotypes.
